# Supplementary material for: Artificial Intelligence Enhances Diagnostic Flow Cytometry Workflow in the Detection of Minimal Residual Disease of Chronic Lymphocytic Leukemia
Source: Cancers (Basel). 2022 May 21;14(10):2537. doi: 10.3390/cancers14102537 (PMC9139233; doi:10.3390/cancers14102537)
Supplement: Supplementary file 1 [file cancers-14-02537-s001.zip › cancers-1728490-supplementary.pdf]

cancers

MDPI

Table S1. Confusion matrix of the F-DNN for cell populations of interest using the test cohort (n = 25,671,598 events from 202 unique cases).

	Ground Truth							
**Inference**	Abnormal CLL cells	Polytypic B cells	Hematogones	Plasma cells	T cells	Aggregates	Debris	Other cell categories
**Abnormal CLL cells**	1,316,803	1330	24	1	257	1255	10,609	63
**Polytypic B cells**	617	195,925	567	0	1	304	5041	52
**Hematogones**	35	529	93,642	57	0	136	4356	185
**Plasma cells**	0	0	13	11,033	1	196	2370	334
**T cells**	367	1	0	0	2,697,327	1908	33,173	2021
**Aggregates**	220	10	7	48	118	173,9028	52,008	97,226
**Debris**	7530	1913	2030	1741	19,013	28,656	3,611,475	124,861
**Other cell categories**	1096	681	743	1358	8344	140,174	154,867	15,297,918

Table S2. Confusion matrix of the L-DNN for cell populations of interest using the test cohort (n = 18,212,266 events from 138 unique cases).

	**Ground Truth**							
**Inference**	**Abnormal CLL cells**	**Polytypic B cells**	**Hematogones**	**Plasma cells**	**T cells**	**Aggregates**	**Debris**	**Other cell categories**
**Abnormal CLL cells**	1358	33	0	0	12	1	125	13
**Polytypic B cells**	38	177,419	461	0	4	191	3658	53
**Hematogones**	5	452	70,908	34	0	35	2874	94
**Plasma cells**	0	3	10	10,749	0	167	1969	277
**T cells**	20	5	2	0	19,43032	1506	20,550	1656
**Aggregates**	0	3	1	24	51	1,213,909	22,833	59,901
**Debris**	146	2060	1632	1731	13,353	18,350	2,585,138	108,669
**Other cell categories**	113	325	445	910	5610	84,854	104,234	11,750,260
